# Contribution of major food companies and their products to household dietary sodium purchases in Australia

**DOI:** 10.1186/s12966-020-00982-z

**Published:** 2020-06-23

**Authors:** Daisy H. Coyle, Maria Shahid, Elizabeth K. Dunford, Cliona Ni Mhurchu, Sarah Mckee, Myla Santos, Barry M. Popkin, Kathy Trieu, Matti Marklund, Fraser Taylor, Bruce Neal, Jason H. Y. Wu

**Affiliations:** 1 The George Institute for Global Health, Faculty of Medicine, UNSW, Level 5, 1 King St Newtown, Sydney, Australia; 2 Department of Nutrition, The University of North Carolina at Chapel Hill, Chapel Hill, USA; 3 National Institute for Health Innovation, The University of Auckland, Auckland, New Zealand; 4 Nielsen, Sydney, Australia; 5 Friedman School of Nutrition Science and Policy, Tufts University, Boston, MA USA

**Keywords:** Sodium, Dietary, Australia, Income, Packaged food, Beverages, Disparities, Sodium intake

## Abstract

**Background:**

The Australian federal government will soon release voluntary sodium reduction targets for 30 packaged food categories through the Healthy Food Partnership. Previous assessments of voluntary targets show variable industry engagement, and little is known about the extent that major food companies and their products contribute to dietary sodium purchases among Australian households.

**Methods:**

The aim of this cross-sectional study was to identify the relative contribution that food companies and their products made to Australian household sodium purchases in 2018, and to examine differences in sodium purchases by household income level. We used 1 year of grocery purchase data from a nationally representative consumer panel of Australian households who reported their grocery purchases (the Nielsen Homescan panel), combined with database that contains product-specific sodium content for packaged foods and beverages (FoodSwitch). The top food companies and food categories were ranked according to their contribution to household sodium purchases. Differences in per capita sodium purchases by income levels were assessed by 1-factor ANOVA. All analyses were modelled to the Australian population in 2018 using sample weights.

**Results:**

Sodium data were available from 7188 households who purchased 26,728 unique products and purchased just under 7.5 million food product units. Out of 1329 food companies, the top 10 accounted for 35% of unique products and contributed to 58% of all sodium purchased from packaged foods and beverages. The top three companies were grocery food retailers each contributing 12–15% of sodium purchases from sales of their private label products, particularly processed meat, cheese and bread. Out of the 67 food categories, the top 10 accounted for 73% of sodium purchased, particularly driven by purchases of processed meat (14%), bread (12%) and sauces (11%). Low-income Australian households purchased significantly more sodium from packaged products than high-income households per capita (452 mg/d, 95%CI: 363-540 mg/d, *P* < 0.001).

**Conclusions:**

A small number of food companies and food categories account for most of the dietary sodium purchased by Australian households. Prioritizing government engagement with these groups could deliver a large reduction in population sodium intake.

## Background

High intake of sodium is a leading dietary risk factor for death and disability globally [1]. Excess dietary sodium intake increases blood pressure, which is a major modifiable risk factor for cardiovascular disease [2] and chronic kidney disease [3]. The World Health Organization (WHO) recognizes the importance of reducing sodium intake and has set a global target of a 30% reduction by 2025, towards the goal of 2 g/d per person (5 g of salt) [4]. Despite this, most populations around the world exceed WHO recommendations, including in Australia, where adults have an estimated average sodium intake of 4 g and 2.9 g for men and women per day, respectively [5].

The WHO promotes reformulation of packaged foods as a priority action area to lower population sodium intake [4]. Concordantly, an increasing number of governments around the world have begun implementing mandatory or voluntary sodium reformulation targets for packaged foods [6]. In Australia, the federal government has drafted voluntary sodium reformulation targets for packaged foods under the Healthy Food Partnership initiative [7]. The Healthy Food Partnership is a public-private partnership that recognizes the importance of engaging with private sector food companies, and the critical role they play in shaping the food system. The Healthy Food Partnership sodium targets are currently being finalized and are soon to be released for 30 categories of packaged foods and beverages [7, 8].

Previous assessments of voluntary nutrient reformulation schemes, including the Food and Health Dialogue (the predecessor of the Healthy Food Partnership) suggest widely varying levels of engagement by food and beverage companies - with some progressing to full reformulation across their product ranges, and others achieving very little [9–11]. Such findings suggest that additional accountability measures are needed to motivate food companies to reformulate their products. A strong approach to transparency and accountability has been effective in areas such as greenhouse gas emissions [12] and tobacco sales [13] – but analogous data on how companies contribute to key nutrients in the food supply is largely lacking.

The primary aims of this study were to use a population-based sample of Australian households who reported their grocery purchases, combined with product-specific sodium information, to 1) identify the relative contribution that different food companies make to household sodium purchases in Australia, and 2) identify the main food categories contributing to sodium purchases by Australians. For policy relevance, analyses were restricted to packaged foods and beverages, as these are the targets of the Healthy Food Partnership. To gain insight into the potential impact of the food supply and product reformulation on disparities in household diets, in secondary analyses we assessed whether household sodium purchases differ according to household income level.

## Methods

This project was approved by the University of New South Wales Human Research Ethics Committee (approval number HC180965).

### Study population

This cross-sectional study used 12 months (January 2018– December 2018) of Nielsen Homescan Consumer Panel data, a commercial dataset that captures grocery purchases, including non-food purchases, made by Australian households. Nielsen Homescan maintains a panel of approximately 10,000 households and these households are recruited through an online application process. To ensure the Homescan panel are representative of Australian households, Nielsen has formed geographical segments with quotas for each geographical area to avoid clustering of households by location. They also control for other factors in the recruitment stage that are relevant to grocery purchasing, including household size, lifestage and income level. To ensure recruited households are demographically and geographically representative of Australian households, the data collected by recruited households are projected to the demographics of the Australia population. Sociodemographic and economic characteristics of the households, including ethnicity and education level of the head of the household, household income and lifestage, and age and sex of all individuals in the household are captured.

### Food and beverage purchase data

Households in Nielsen Homescan are provided with handheld barcode scanners to record foods and beverages brought into the home from all retail outlets including supermarkets, grocers, convenience stores and pharmacies. Data on non-barcoded items such as unpackaged fruit, vegetables and deli meats are collected using standard barcodes within a scanning guide booklet provided by Nielsen. Information on all food and beverage purchases made throughout the year are reported by households and this data is collected on a weekly basis. Data are not collected on food purchased and consumed outside of the home. In Australia, approximately two thirds of all food and beverage expenditure (excluding alcohol) occurs at supermarkets (Nielsen 2019, personal communication, July 2019) [14]. To capture regular shopping habits throughout the year and to account for products that are stored and not consumed immediately, we used year-level purchase data by summing all food and beverage purchases made during the calendar year.

### Household eligibility and exclusion criteria

Household eligibility was based on standard criteria provided by Nielsen. To be included in the current analyses, households must have been on the panel for the entire 52-week time frame and reported purchase data (at least one barcode per week) for at least 50% of the weeks. Household data were excluded from analyses if they were missing any demographic information or if Nielsen thresholds for expenditure on all purchases (food and non-foods) were not met (≥$5 on average for each week over the time frame, i.e. at least $260 per household over the 52-week period). To account for households possibly under-reporting purchase information for foods and beverages, we further excluded households with the lowest annual food and beverage expenditure (< 2.5th percentile defined separately for single-member households and multi-member households).

### Food and beverage nutrient data

The product-specific sodium content of foods purchased was obtained from the 2018 FoodSwitch Annual Database [15]. This database contains nutrition information obtained directly from the mandatory Nutrition Information Panel (NIP) of all packaged foods and beverages available for sale from five large supermarket retailers in Sydney, Australia (Woolworths, Coles, Aldi, IGA and Harris Farm) during the months of August to November in 2018.

### Food company classification

As food companies may own subsidiary companies (brands), Nielsen has mapped out the ownership of subsidiary companies by parent companies using internet searches, internal consistency checks and direct contact with food companies. These ownership structures are updated regularly to ensure any changes due to company acquisitions are captured correctly. Therefore, all food companies analyzed in this paper are reported at the parent company level. For example, foods and beverages branded under ‘Pepsi’, ‘Lays’, ‘Doritos’, ‘Lipton’ and ‘Gatorade’ are reported under the parent company of ‘Pepsico’. For the purposes of this paper, we further identified which food companies were ‘retailers’. These are supermarket retailers that sell their own ‘private-label’ products, also known as ‘own brand’, ‘store brand’, ‘generic’ or ‘home brand’ products, exclusively in their own stores [16].

### Merging Nielsen Homescan and FoodSwitch databases

Steps taken to match foods and beverages in the Nielsen Homescan and FoodSwitch database are outlined in Additional Figure 1 and described below**.**

#### Exclusion of products not relevant for analyses

We first excluded non-food and beverage products from the Nielsen Homescan database, such as medicinal items and cleaning products, as well as any food and beverages sold unpackaged such as fruits, vegetables, store-prepared bakery items and ready-to-eat dishes, as these products are not targeted for reformulation by the Healthy Food Partnership. Variety packs with multiple NIPs and products were also excluded as these cannot be easily categorized. The same exclusion criteria were applied to products in the FoodSwitch database.

#### Matching products across databases

Eligible households purchased a total of 59,406 unique food and beverages relevant for our analyses, with a total quantity (i.e. number of units sold) of ~ 8.4 million units. Initial matching to FoodSwitch was carried out using unique barcodes associated with each product. Out of the 59,406 unique products, 22,998 (39%) were matched to products in FoodSwitch, which accounted for 84% of the quantity of product units (*n* = 7,106,179) purchased by the households. To further improve our coverage of the products purchased by the Nielsen Homescan panel, we followed the methods developed by Slining et al. [17], which enabled additional matching using 1) product name, 2) product name following removal of irrelevant descriptors from the product name, and 3) applying sodium values to single ingredient foods such as honey, eggs and oils, using the category mean from FoodSwitch. Using these methods, the number of unique products in the Nielsen Homescan database matched to FoodSwitch increased to 26,728 (45%) representing ~ 7.5 million units (89%) of the quantity of products purchased in 2018 (Additional Figure 1). Non-matched products were not concentrated in any particular food category. The food categories with the largest volume of unmatched products included bread (5% of all unmatched products), snack foods (5%), biscuits and cookies (4%), and herbs and spices (4%).

### Statistical analysis

All statistical analyses were conducted using Stata 15.1 (StataCorp). Two-sided *p* < 0.05 was considered statistically significant.

We assessed Australian household sodium purchases from packaged foods and beverages using three outcome measures 1) sodium per capita, the amount of sodium in milligrams (mg) purchased daily per person and 2) sodium density, the amount of sodium relative to the energy content of products (mg/1000 kcal), and 3) the purchase-weighted sodium content (mg/100 g), the weight of sodium (mg) divided by the total weight (g) of products purchased (package size x quantity sold in 2018). For sodium density, the number of households that had an optimal sodium density of ≤1100 mg/1000 kcal was also assessed. This is based on the recommended daily sodium intake in the Dietary Approaches to Stop Hypertension (DASH) diet – representing 2300 mg of sodium per day or less for an average 2100 cal/day diet [18, 19].

The major food companies and food categories contributing to Australian household purchases of sodium were identified and ranked according to their relative (%) contribution to total sodium purchases. Descriptive data were presented for the top 10 food companies and food categories, with the remainder grouped and reported as an “other” category. We further identified the top three food categories that contributed the most to household purchases of sodium for each of the top 10 food companies as well as the proportion of products from each top 10 food companies that met the proposed Healthy Food Partnership sodium targets. Food products were grouped according to the FoodSwitch categorization structure, which uses a hierarchical system, classifying products into food groups (e.g. bread and bakery products) and categories (e.g. bread) and subcategories (e.g. white bread). A full list of food categories included in the analyses are provided in Additional Table 1.

In secondary analyses, we explored differences in household sodium purchases by household income level. Households were classified as low, middle or high-income based on percentile cut-offs as defined by the Australian Bureau of Statistics (ABS) Australian Household Income and Wealth survey data in 2015–16 [20]. Mean sodium purchases per capita were calculated by dividing total household sodium purchases by the number of people within the household. Differences in mean sodium purchases per capita were assessed using 1-factor ANOVA test with Tukey honest significance difference (HSD) test post-hoc analyses.

All analyses were modelled to the Australian population in 2018 using sample weights to ensure the data accurately represented household purchasing habits in the Australian population. Sample weights were provided by Nielsen and were based on Australian census data pertaining to household size, location, lifestage and income [21].

To estimate the total sodium acquired from all grocery purchases (packaged and unpackaged foods and beverages), sensitivity analyses were conducted on all barcoded and non-barcoded items (e.g. unpackaged fruit, vegetables, breads and deli meats) collected as part of Nielsen scanning guide. The sodium content of non-barcoded products were obtained from the AUSNUT 2011–13 food nutrient database [22].

To explore the potential influence of under-reporting, we also conducted further sensitivity analysis excluding households in the ≤5th percentile for annual food and beverage expenditure, with the percentile value defined separately for single and multi-member households.

## Results

### Household characteristics

Of the 11,056 households in the Nielsen Homescan panel in 2018, 3868 were excluded for not meeting eligibility criteria, leaving 7188 households for the analyses. Compared with the most recent Australian census data, Nielsen households had similar household size and household location characteristics, although, they had a slightly higher proportion of low-income households (Additional Table 2). However, when households were modelled to the Australian population, the proportions closely aligned with the 2016 census data across each of the household characteristics.

### Amount of sodium purchased per capita per day

The mean ± SE total sodium acquired from packaged foods and beverages in 2018 was 1443 ± 0.3 mg/day per capita (~ 3.6 g salt, Table 1). This was predominately attributed to purchases of food (1059 mg/day, 74% of total sodium purchases), followed by table salt (311 mg/d, 21%) and beverages (73 mg/d, 5%). The sodium density of households’ total packaged food and beverage purchases was 1466 ± 89 mg/1000 kcal. About a quarter (27%) of Australians had total packaged food and beverage purchases with optimal sodium density (≤1100 mg/1000 kcal).

**Table 1:** Sodium acquired from Australian households packaged food and beverage purchases

Category	Weight of products purchased (g/d per capita) Mean^**a**^	Sodium (mg/d per capita)		Contribution to total weight of sodium purchases (%)
		Mean^**1**^	Median (25th to 75th percentiles)	
Foods	354	1059	960 (656–1334)	74
Beverages	280	73	56 (29–97)	5
Table salt	1	311	79 (0–380)	21
Total	636	1443	1253 (847–1793)	100

^a^Standard error (SE) for weight of products purchased (g/d per capita) and sodium (mg/d per capita) not displayed as SE ≤0.3 for each mean value

### Contribution of food companies to sodium purchases

A total of 1329 food companies contributed to packaged foods and beverages purchased by Australian households. Among these, the top 10 companies contributed to a total of 58% of all sodium purchased, despite accounting for only 35% of products (Table 2). The three largest contributors were retailers, each contributing between 12 and 15% of all sodium purchased. For these retailers, the total weight of products sold ranged between 96 and 116 g/d per capita, substantially higher than other companies ranked within the top 10, which ranged from 6 to 31 g/d. The purchase-weighted sodium content ranged from 267 to 386 mg/100 g for the top three retailers, lower than nearly all other companies ranked within the top 10, which ranged from 321 to 581 mg/100 g.

**Table 2:** Characteristics and contributions of the top 10 food companies contributing to Australian household purchases of sodium from packaged foods and beverages

Company rank^**1**^	No. of unique products	Total weight of products purchased (g/d per capita) Mean^**2**^	Sodium (mg/d per capita)		Mean purchase-weighted sodium content (mg/100 g)^**3**^	Contribution to total weight of sodium purchases	
			Mean^**2**^	Median (25th to 75th percentiles)		Total (%)	Top 3 food categories contributing to sodium purchases^**4**^
1 (Retailer)	2406	116	245	95 (19–331)	386	15	Processed meat (19%); Cheese (14%); Bread (11%)
2 (Retailer)	2313	96	158	78 (20–205)	302	12	Processed meat (19%); Cheese (15%); Bread (14%)
3 (Retailer)	2317	103	141	77 (23–186)	267	12	Processed meat (17%); Bread (17%); Cheese (15%)
4	163	10	47	26 (10–59)	581	4	Bread (80%); Processed meat (18%); Cakes, muffins and pastries (2%)
5	268	11	42	22 (9–51)	505	3	Bread (62%); Mayonnaise and salad dressings (15%); Cakes, muffins and pastries (7%)
6	536	14	37	25 (11–48)	321	3	Vegetables (34%); Sauces (29%); Processed fish (22%)
7	174	7	34	21 (8–43)	434	3	Biscuits/cookies (98%); Crisps and snacks (2%)
8	535	6	26	15 (5–33)	448	2	Sauces (79%); Herbs and spices (10%); Chocolate and sweets (6%)
9	432	7	26	16 (6–33)	359	2	Spreads and dips (58%); Chocolate and sweets (13%); Biscuits/cookies (13%)
10	216	31	23	13 (5–27)	492	2	Crisps and snacks (74%); Soft drinks (14%); Biscuits/cookies (7%)
Others	17,368	303	490	429 (271–637)	383	42	

^1^Rank = Companies are ranked in order of their contribution to the total weight of sodium purchased by Australian households, from highest to lowest. Results for the top 10 companies are shown separately, with the remaining 1319 companies summed together to simplify data presentation. ^2^Standard error (SE) for mean weight of products purchased (g/d per capita) and sodium (mg/d per capita) not displayed as SE ≤0.1 for each mean value. ^3^Purchase-weighted sodium content (mg/100 g): weight of sodium (mg) divided by the total weight (g) of products purchased (package size x quantity sold in 2018). ^4^% contribution of each of the top 3 food categories were calculated as a total of all sodium purchases within each company

Across the top three retailers, the majority of sodium purchased derived from processed meat (17–19%), cheese (14–15%) and bread (11–17%) (Table 2). Across the remaining top 10 food companies, there was a diverse range of food categories that contributed most to sodium purchases, including cakes, muffins and pastries (2–7%), biscuits/cookies (7–98%), chocolate and sweets (6–13%), sauces (29–79%) and crisps and snacks (2–74%). Across the top 10 companies, 49% of their products purchased by the households met the proposed Healthy Food Partnership sodium targets although this was highly variable across companies ranging from 8 to 82% (Additional Figure 2).

### Contribution of food categories to sodium purchases

Across 67 packaged food and beverage categories examined, the top 10 food category sources of sodium together contributed to 73% of sodium in household purchases **(**Table 3**).** On average, the largest contributors to sodium purchases were processed meat (152 mg/d per capita, 14% of total sodium purchases), bread (130 mg/d per capita, 12%), sauces (125 mg/d per capita, 11%) and cheese (111 mg/d per capita, 10%). Within the top 10 categories, the total weight of products sold ranged from 8 g/d for crisps and snacks to 125 g/d for milk (Table 3). The purchase-weighted sodium content was highly variable across food categories, ranging from 45 mg/100 g for milk to 986 mg/100 g for sauces (Table 3). Out of the top 10 food categories, four did not have a proposed Healthy Food Partnership sodium target available despite their considerable contribution to sodium purchases: processed vegetables (6%), milk (5%), edible oils (4%), and spreads and dips (3%) (Table 3).

**Table 3:** Major packaged food and beverage categories contributing to Australian household purchases of sodium

Food category rank^**1**^	Food category	Total weight of products purchased (g/d per capita) Mean^**2**^	Sodium (mg/d per capita)		Mean purchase-weighted sodium content (mg/100 g)^**3**^	Contribution to total weight of sodium purchases (%)	HFP proposed target (Yes/No)
			Mean^**2**^	Median (25th to 75th percentiles)			
1	Processed meat	26	152	111 (53–198)	703	14	Yes
2	Bread	31	130	103 (54–176)	451	12	Yes
3	Sauces	16	125	99 (54–161)	986	11	Yes
4	Cheese	15	111	86 (49–144)	736	10	Yes
5	Processed vegetables	40	66	42 (20–80)	212	6	No
6	Biscuits/cookies	14	59	43 (22–77)	422	5	Yes
7	Milk	125	55	39 (18–75)	45	5	No
8	Crisps and snacks	8	46	32 (15–60)	633	4	Yes
9	Edible oils	10	45	30 (14–58)	409	4	No
10	Spreads and dips	6	39	27 (13–49)	730	3	No
	Others	347	317	272 (181–398)	218	27	–

^1^Rank = Food categories are ranked in order of their contribution to the total volume of sodium purchased by Australian households, from highest to lowest. Results for the top 10 food categories are shown separately, with the remaining 57 food categories summed together to simplify data presentation. ^2^Standard error (SE) for weight of products purchased (g/d per capita) and sodium (mg/d per capita) not displayed as SE ≤0.1 for each mean value. HFP, Healthy Food Partnership. ^3^Purchase-weighted sodium content (mg/100 g): weight of sodium (mg) divided by the total weight (g) of products purchased (package size x quantity sold in 2018)

### Sodium purchases according to household income

**Fig. 1:**
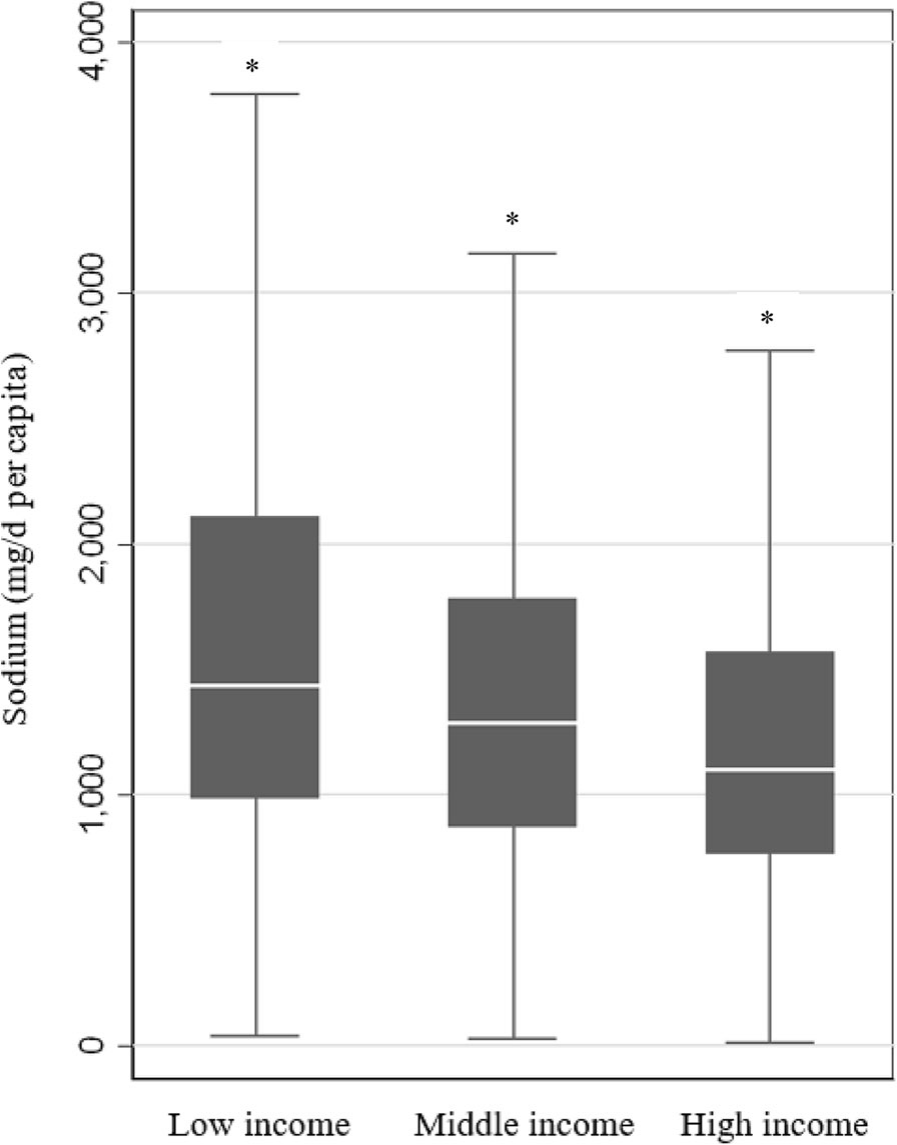
Sodium (mg/d per capita) acquired by Australian households from packaged food and beverage purchases according to income level. Low income = $954 per week or less per household, Middle = $955 - $2000 per week per household, High = $2469 per week per household. The box displays the interquartile range, and the median value is marked as the line within the box. Whiskers extend to the lowest datum within 1.5 interquartile range of the lower quartile, and the highest datum within 1.5 IQR of the upper quartile. Low income households had the highest sodium purchases per capita. *indicates a significant difference across all income levels (*P* < 0.01)

Low-income households had significantly higher per capita sodium purchases than middle-income households (mean difference, 256 mg/d, 95%CI: 164-345 mg/d, *P* < 0.001) and high-income households (452 mg/d, 95%CI: 363-540 mg/d, *P* < 0.001) (Fig. 1). Low-income households also purchased significantly higher per capita weight of packaged food and beverages compared with middle-income (111 g/d, 95%CI: 85-138 g/d, *P* < 0.001) and high-income households (216 g/d, 95%CI: 190-243 g/d, *P* < 0.001). The purchase-weighted sodium content was similar across all income groups ranging from 479 to 481 mg/100 g for total purchases of packaged foods, beverages and table salt (Additional Table 3).

### Sensitivity analyses

Inclusion of non-barcoded foods and beverages increased sodium purchase estimates by 15% to 1683 ± 0.4 mg/day per capita (~ 4.1 g salt, Additional Table 4). Inclusion of these additional items also increased the weight of food and beverages purchases by 36% to 868 ± 0.2 g/day per capita. The majority of sodium purchased from non-barcoded items came from processed meat (84% of all sodium purchased from non-barcoded foods), bread (5%) and vegetables (4%).

Excluding households in the lowest 5th percentile for total annual spend for foods and beverages (resulting in *n* = 7004 households remaining in the analyses) did not appreciably change sodium estimates (mg/d per capita) or percentage contribution of sodium from foods, beverages and table salt (results not shown).

## Discussion

Our study quantified the relative contribution that food companies and their products make to household sodium purchases in Australia. Products from just a small number of food companies accounted for a large amount of the total sodium purchased, with the majority of sodium purchased from processed meat, bread, sauces and cheese. Most households had packaged food and beverage purchases without optimal sodium density. Lower income households in Australia were found to purchase significantly higher amounts of sodium from packaged foods and beverages compared to middle- and higher-income households.

A key finding from our analyses is that packaged foods and beverages produced by just 10 food companies (representing 0.7% of total number of companies) accounted for over half of all sodium purchases. This was largely attributable to the private-label products of three major supermarket retailers and was primarily driven by the large volume of these products purchased by Australian households, rather than a higher sodium content. These findings suggest that small reductions in the sodium content across key food categories and companies has considerable potential to create meaningful change to population sodium intake levels, and that the Australian government should consider prioritizing engagement efforts toward key companies.

Another important finding is that at present, only six of the top 10 food categories that contribute most to household sodium purchases actually have reformulation targets defined by the Healthy Food Partnership [8]. For some products, such as plain milk, reformulation targets would not be appropriate given these products do not contain added sodium. However, even assuming 100% adoption of the Healthy Food Partnership targets across all companies, it has been estimated this would only achieve an − 9% reduction in sodium intakes (− 212 mg/person/day) [8]. Our findings reinforce the need for broadening the scope of the Healthy Food Partnership by adding further category-specific targets. In particular, our analyses suggest additional reformulation targets should be set for some processed vegetables (e.g. pickled vegetables), edible oils (e.g. butter and margarine), and spreads and dips. Although some of these foods may be consumed in only small amounts each day, they are high in sodium and are consumed frequently across the population. Therefore, reducing the sodium content of these foods could still contribute to a meaningful reduction in sodium across the population. Previous analyses indicate that the sodium content of packaged foods varies extensively across the even quite similar foods [10, 23, 24] indicating that reformulation is likely feasible from both a food technology and customer taste perspective [25]. Furthermore, the UK government has already set sodium targets for these three categories [26], demonstrating the potential scope of a broader target range in Australia.

Our study also found that just a small number of food categories disproportionately accounted for total sodium purchases. This finding aligns with existing literature conducted in the US [27] and the UK [28]. In the US, the top contributors to sodium purchases in 2014 were condiments/sauces/dips, mixed dishes, salty snacks, breads and processed meats [27] and in the UK, processed meat, bread, dairy products and sauces and spreads [28]. While these findings are not directly comparable due to different classification systems used for categorizing products, they still highlight a number of key food categories globally that consistently contribute to a large share of sodium purchases.

Prior research in Australia and globally has also demonstrated higher sodium intakes in individuals with a lower socio-economic status (SES) [29–32]. By using contemporary and objectively collected sodium purchase data, representative of Australian households, our findings suggest that differences in sodium consumption according to SES is likely at least partly driven by larger volumes of packaged foods and beverages purchased by lower-income households, rather than due to purchases of higher-sodium content products. Assuming purchasing patterns of packaged foods were to remain stable in the population, our findings suggest that reformulation of packaged products to a lower sodium content will likely result in greater proportional reduction in sodium intake in lower-income households, which could contribute to a reduction in cardiovascular disease-related health disparity [33].

A key strength of this study was the use of objective purchase data to assess household packaged food and beverage purchases in a nationwide sample of Australian households, which was comparable to the average Australian household size, household income and location. The use of continuously collected purchase information with brand- and product-specific nutrient data is an innovative approach to accurately and objectively estimate the contribution of major food companies and their products to household purchases of sodium. Furthermore, our findings at the food company and food category level provide detailed baseline data to allow us to track long-term trends in sodium purchases and monitor the impact of the Healthy Food Partnership on sodium reductions in the Australian food supply [34].

A limitation of the analyses is that under-reporting of purchases by the Nielsen Homescan panel is likely, with previous research suggesting under-reporting rates of 10–20% [35, 36], although we attempted to control for such underreporting by excluding households below spending threshold limits, which did not have a discernable impact on our findings. As we achieved an 89% match rate between FoodSwitch and the purchase quantity of products in the Nielsen dataset, our results further slightly underestimated true household sodium purchases. However, such under-estimates are unlikely to have affected the validity of our results as the main results of our paper involved ranking companies and food categories by their relative contribution. Given our study analyzed packaged food and beverages available in Australia and purchased by Australian households, our findings may not be generalizable to other countries. This study did not assess household food and beverage expenditure outside of the home, which is a growing portion of household food spending, and represents an important area for future research to understand population sodium exposure [37].

## Conclusions

In conclusion, in this nationally representative sample of Australian households, a small number of food companies and their products accounted for the majority of sodium purchases, and sodium acquired from packaged foods was highest for low-income households. There is considerable potential for a select group of food companies to reduce their sodium levels in line with the proposed Healthy Food Partnership targets to reduce population sodium intake, and this would likely have greatest impact on the most disadvantaged households in the Australian population.

## Supplementary information

**Additional file 1:Additional Table 1.** Food categorisation system and categories included in the current analyses. **Additional Figure 1.** Merging of FoodSwitch and Nielsen Homescan datasets. **Additional Table 2.** Sociodemographic characteristics of the Nielsen Homescan Consumer Panel in 2018 projected to the Australian population compared with 2016 Australian Census data. **Additional Figure 2.** Percentage of products meeting and not meeting proposed Healthy Food Partnership sodium targets. **Additional Table 3.** Sodium from Australian household packaged food and beverage purchases according to income level. **Additional Table 4.** Sodium acquired from Australian household food and beverage purchases, including barcoded and non-barcoded products.

## Data Availability

The data that support the findings of this study are available from Nielsen and FoodSwitch, but restrictions apply to the availability of these data, which were used under license for the current study, and so are not publicly available.

## Abbreviations

WHO: World health organization
NIP: Nutritional information panel
DASH: Dietary approaches to stop hypertension
ABS: Australian bureau of statistics
HSD: Honest significance test
SES: Socioeconomic status
