# Correction to: Contribution of major food companies and their products to household dietary sodium purchases in Australia

**DOI:** 10.1186/s12966-020-01064-w

**Published:** 2020-12-04

**Authors:** Daisy H. Coyle, Maria Shahid, Elizabeth K. Dunford, Cliona Ni Mhurchu, Sarah Mckee, Myla Santos, Barry M. Popkin, Kathy Trieu, Matti Marklund, Fraser Taylor, Bruce Neal, Jason H. Y. Wu

**Affiliations:** 1 The George Institute for Global Health, Faculty of Medicine, UNSW, Level 5, 1 King St Newtown, Sydney, Australia; 2 Department of Nutrition, The University of North Carolina at Chapel Hill, Chapel Hill, USA; 3 National Institute for Health Innovation, The University of Auckland, Auckland, New Zealand; 4 Nielsen, Sydney, Australia; 5 Friedman School of Nutrition Science and Policy, Tufts University, Boston, MA USA

**Correction to: Int J Behav Nutr Phys Act 17, 81 (2020)**

**https://doi.org/10.1186/s12966-020-00982-z**

Following publication of the original article [1], the authors identified errors in Table 2 and Table 3. The correct tables are given below.

**Table 2:** Characteristics and contributions of the top 10 food companies contributing to Australian household purchases of sodium from packaged foods and beverages

Company rank^**1**^	No. of unique products	Total weight of products purchased (g/d per capita) Mean^**2**^	Sodium (mg/d per capita)		Mean purchase-weighted sodium content (mg/100 g)^**3**^	Contribution to total weight of sodium purchases	
			Mean^**2**^	Median (25th to 75th percentiles)		Total (%)	Top 3 food categories contributing to sodium purchases^**4**^
1 (Retailer)	2406	74	156	15 (0–166)	386	15	Processed meat (19%); Cheese (14%); Bread (11%)
2 (Retailer)	2313	84	138	56 (9–180)	302	12	Processed meat (19%); Cheese (15%); Bread (14%)
3 (Retailer)	2317	94	128	65 (14–169)	267	12	Processed meat (17%); Bread (17%); Cheese (15%)
4	163	9	42	21 (6–53)	581	4	Bread (80%); Processed meat (18%); Cakes, muffins and pastries (2%)
5	268	10	39	19 (7–47)	505	3	Bread (62%); Mayonnaise and salad dressings (15%); Cakes, muffins and pastries (7%)
6	536	14	36	24 (10–47)	321	3	Vegetables (34%); Sauces (29%); Processed fish (22%)
7	174	7	32	18 (6–41)	434	3	Biscuits/cookies (98%); Crisps and snacks (2%)
8	535	7	26	16 (6–32)	448	2	Sauces (79%); Herbs and spices (10%); Chocolate and sweets (6%)
9	432	5	25	14 (4–32)	359	2	Spreads and dips (58%); Chocolate and sweets (13%); Biscuits/cookies (13%)
10	216	28	21	11 (4–25)	492	2	Crisps and snacks (74%); Soft drinks (14%); Biscuits/cookies (7%)
Others	17,356	303	490	429 (271–637)	383	42	

^1^Rank = Companies are ranked in order of their contribution to the total weight of sodium purchased by Australian households, from highest to lowest. Results for the top 10 companies are shown separately, with the remaining 1319 companies summed together to simplify data presentation. ^2^Standard error (SE) for mean weight of products purchased (g/d per capita) and sodium (mg/d per capita) not displayed as SE ≤0.1 for each mean value. ^3^Purchase-weighted sodium content (mg/100 g): weight of sodium (mg) divided by the total weight (g) of products purchased (package size x quantity sold in 2018). ^4^% contribution of each of the top 3 food categories were calculated as a total of all sodium purchases within each company

**Table 3:** Major packaged food and beverage categories contributing to Australian household purchases of sodium

Food category rank^**1**^	Food category	Total weight of products purchased (g/d per capita) Mean^**2**^	Sodium (mg/d per capita)		Mean purchase-weighted sodium content (mg/100 g)^**3**^	Contribution to total weight of sodium purchases (%)	HFP proposed target (Yes/No)
			Mean^**2**^	Median (25th to 75th percentiles)			
1	Processed meat	26	148	108 (49–194)	703	14	Yes
2	Bread	31	129	102 (53–175)	451	12	Yes
3	Sauces	16	124	98 (53–160)	986	11	Yes
4	Cheese	15	110	85 (48–143)	736	10	Yes
5	Processed vegetables	40	66	41 (20–80)	212	6	No
6	Biscuits/cookies	14	58	43 (22–76)	422	5	Yes
7	Milk	124	54	39 (18–74)	45	5	No
8	Crisps and snacks	7	44	30 (13–59)	633	4	Yes
9	Edible oils	10	43	30 (13–57)	409	4	No
10	Spreads and dips	6	38	26 (11–48)	730	3	No
	Others	347	317	272 (181–398)	218	27	–

^1^Rank = Food categories are ranked in order of their contribution to the total volume of sodium purchased by Australian households, from highest to lowest. Results for the top 10 food categories are shown separately, with the remaining 57 food categories summed together to simplify data presentation. ^2^Standard error (SE) for weight of products purchased (g/d per capita) and sodium (mg/d per capita) not displayed as SE ≤0.1 for each mean value. HFP, Healthy Food Partnership^. 3^Purchase-weighted sodium content (mg/100 g): weight of sodium (mg) divided by the total weight (g) of products purchased (package size x quantity sold in 2018)

The original article [1] has been corrected.
