# Supplementary material for: Contribution of major food companies and their products to household dietary sodium purchases in Australia
Source: Int J Behav Nutr Phys Act. 2020 Jun 23;17:81. doi: 10.1186/s12966-020-00982-z (PMC7310483; doi:10.1186/s12966-020-00982-z)
Supplement: Supplementary file 1 — Additional file 1:Additional Table 1. Food categorisation system and categories included in the current analyses. Additional Figure 1. Merging of FoodSwitch and Nielsen Homescan datasets. Additional Table 2. Sociodemographic characteristics of the Nielsen Homescan Consumer Panel in 2018 projected to the Australian population compared with 2016 Australian Census data. Additional Figure 2. Percentage of products meeting and not meeting proposed Healthy Food Partnership sodium targets. Additional Table 3. Sodium from Australian household packaged food and beverage purchases according to income level. Additional Table 4. Sodium acquired from Australian household food and beverage purchases, including barcoded and non-barcoded products. [file 12966_2020_982_MOESM1_ESM.docx]

**Additional Table 1. Food categorisation system and categories included in current analyses**

| **Food group** | **Food category** | **Food subcategories** |
| --- | --- | --- |
| **Bread and bakery products** | Biscuits and cookies | Filled and unfilled sweet biscuits |
|  | Bread | White, wholemeal and mixed grain/seed sliced bread and bread rolls, fruit bread and fruit-based muffins/rolls, wraps, Turkish bread, bagels, crumpets, pizza bases and flatbreads |
|  | Cakes, muffins and pastries | Scones, pikelets, doughnuts, sweet pastries, cakes, crepes, slices, cake, pikelet and pancake dry mixes |
| **Cereal and grain products** | Breakfast cereals | Ready to eat breakfast cereals, oats and other breakfast cereals that require heating |
|  | Cereal and nut-based bars | Plain, chocolate-topped and yoghurt-topped cereal-based bars |
|  | Couscous | Flavoured and plain couscous |
|  | Noodles | Savory, flavored and plain noodles and noodle-based dishes |
|  | Pasta | Canned, fresh, dry and packet pasta |
|  | Rice | Savory, flavored and plain rice |
|  | Other cereal and grain products | Flour and unprocessed cereals |
| **Confectionery** | Chewing gum | Sugar-sweetened and sugar-free chewing gums |
|  | Chocolate and sweets | Chocolate-based confectionery and sugar-based confectionery |
|  | Cough lollies |  |
|  | Jelly |  |
| **Convenience foods** | Meal kits | Kits with ingredients to put meals together |
|  | Other frozen foods | Other pre-prepared frozen foods not included in other categories |
|  | Pizza | Frozen and refrigerated pre-prepared pizzas |
|  | Pre-prepared salads and sandwiches | Chilled pre-prepared salads, sandwiches and sushi |
|  | Ready meals | Frozen, chilled and ambient pre-prepared meals |
|  | Soup | Canned, chilled and ambient soups and soup mixes |
| **Dairy** | Cheese | Feta, haloumi, parmesan and other high-salt cheeses, processed cheese slices, cheddar cheeses (including shredded, block or sliced), soft cheeses such as cream cheese, ricotta and cottage cheese |
|  | Cream | Thickened, sour and regular cream products |
|  | Desserts | Dairy-based desserts and dessert mixes |
|  | Ice cream and edible ices | Dairy and non-dairy-based ice cream varieties and edible ices |
|  | Milk | Flavored and unflavored dairy milks, soy and plant-based milks, and condensed, evaporated and powdered milk products |
|  | Yoghurt and yoghurt products | Fruit, flavored and natural yoghurts including yoghurt drinks |
| **Edible oils and oil emulsions** | Coconut oil |  |
|  | Cooking oil sprays |  |
|  | Cooking oils | Olive, canola, vegetable and other cooking oils |
|  | Edible oils | Margarine, salted butter and unsalted butter |
| **Eggs** | Eggs |  |
| **Fish and fish products** | Processed fish | Plain and flavored canned seafood, chilled processed fish products, coated and uncoated fish products |
| **Fruit and vegetables** | Fruit | Dried fruit products including coconut, fruit-based bars, fruit products canned in juice or syrup, fruit gels, fruits in jelly and fruit puree |
|  | Herbs and spices | Curry powder, herb pastes, herbs, salt, seasoning, seeds and spices |
|  | Jam and marmalades | Jams, marmalades, fruit spreads and other preserves |
|  | Nuts and seeds | Salted and unsalted nuts and seeds |
|  | Processed vegetables | Canned and pickled vegetables, frozen vegetables and potato-based products, dried vegetables, fresh packaged fruit and vegetables |
| **Meat and meat alternatives** | Meat alternatives | Meat-free products, plain tofu and other meat-free alternatives |
|  | Processed meat | Pre-packaged bacon, sliced deli meats, salami and cured meats, sausages and hot dogs, meat burgers, canned meat products, frozen and chilled meats including meat pies and sausage rolls, whole hams, pate and meat spreads |
| **Beverages** | Beverage mixes |  |
|  | Coffee and tea | All coffee and tea products |
|  | Cordials | Sugar-free cordials and sugar-sweetened cordials |
|  | Electrolyte drinks | Sugar-free and sugar-sweetened sports electrolyte drinks |
|  | Energy drinks | Sugar-free and sugar-sweetened energy drinks |
|  | Fruit and vegetable juices | Fresh and ambient fruit and vegetable juices |
|  | Soft drinks | Sugar-free soft drinks and sugar-sweetened soft drinks |
|  | Waters | Plain and flavoured waters |
| **Sauces, dressings, spreads and dips** | Mayonnaise and salad dressings | Mayonnaise and salad dressings (oil-based and vinegar-based) |
|  | Sauces | Asian sauces including soy, fish, oyster sauce, ambient and fresh pasta sauces, gravies and stock, mustards, marinades and recipe bases, meat accompaniments, plain and flavored tomato paste products, steak, HP and Worcestershire sauces |
|  |  |  |
|  |  |  |
|  | Spreads and dips | Chilled and ambient dips and salsa, relishes, chutneys and pickles, peanut butter and other nut spreads, savory spreads, sweet spreads, yeast-extract spreads (e.g. vegemite) |
| **Snack foods** | Crisps and snacks | Plain and flavored potato crisps, extruded snacks, vegetable based snacks, plain and flavored corn chips, pretzels, popcorn and other snack foods |
| **Special foods** | Baby foods | Baby food and formula |
|  | Breakfast beverages |  |
|  | Diet drink mixes (meal replacements) |  |
|  | Milk-based protein drinks |  |
|  | Other fitness or diet products |  |
|  | Protein and diet bars |  |
|  | Sports gels |  |
|  | Sports/protein powders |  |
| **Sugars, honey and related products** | Condensed caramel |  |
|  | Dessert additions |  |
|  | Dessert toppings |  |
|  | Honey |  |
|  | Sugar |  |
|  | Sweeteners |  |
|  | Syrup |  |

Blank cells indicate no subcategories

**Households excluded (n=3,868)**

**Unique products excluded
(n =40, 828)**

- Non-food and beverage items
  (n = 35,495)
- Unpackaged food and beverage items (n = 226)
- Alcohol (n = 2,636)
- Vitamins and health supplements (n=2,471)

**2018 FoodSwitch Monitoring Dataset (MD)**Unique food and beverage products (n=28,369)

**Unique products excluded (n=691)**

- Products unable to categorised (n = 42)
- Alcohol (n = 159)
- Vitamins and health supplements (n=490)

**Unique foods/beverage products from FoodSwitch MD**
(n=27,678)

**1-year of Nielsen Homescan cleaned data:**
Households: n = 7,188
Unique food/beverage products: n= 59,267
Quantity sold: n= 8,350,738

**1-year of Nielsen Homescan data:**
Households: n=11,056
Unique food and beverage products: n=100,095

**Match step 1: Direct match by barcode**

Unique products: n = 22,988 (39%); Quantity sold: n = 7,047,117 (84%)

**Match step 2**: **Direct match by product name**
Unique products: n = 2,546 (4.3%); Quantity sold: n = 294,363 (3.5%)

**Match step 3:** **Direct match by removal of nutritionally irrelevant descriptors**(i.e. container type, shape of the product)
Unique products: n = 42 (0.1%); Quantity sold: n = 12,659 (0.2%)

**Match step 4: Sodium imputation for single-ingredient products** **(eggs, honey and oils) with a missing sodium value**
The imputed value was the mean sodium value for each of the three categories from FoodSwitch.
Unique products: n = 1,140 (1.9%); Quantity sold: n = 52,320 (0.6%)

**Total products included in the final analysis**

Unique products: n = 26,716 (45%); Quantity sold: n = 7,406,459 (89%)

**Additional Figure 1. Merging of FoodSwitch and Nielsen Homescan datasets.** Households were excluded from analyses based on eligibility criteria provided by Nielsen. To be included in the analyses, households must have been on the panel for the entire 12-month time frame and reported purchase data (at least one barcode per week) for at least 50% of the weeks. Households data were deemed unreliable and excluded from analyses if they were missing any demographic information or if thresholds for expenditure were not met (≥$5 on average for each week over the time frame, i.e. at least $260 per household over the 52-week period. To account for households possibly under-reporting purchase information for foods and beverages, we further excluded households with the lowest annual food and beverage expenditure (<2.5^th^ percentile for single-member households and multi-member households). Products were excluded from both the Nielsen Homescan and FoodSwitch databases if they were not relevant for analyses. This included non-food and beverage products, such as medicinal items and cleaning products, as well as any food and beverages sold unpackaged such as fruits, vegetables, store-prepared bakery items and ready-to-eat dishes, as these products are not required to display a NIP and are not targeted for reformulation by the HFP. Variety packs with multiple NIPs and products were also excluded as these cannot be categorised. The databases were merged by matching products according to barcode (Step 1) followed by additional matching steps to increase match rate (Steps 2-4).

**Additional Table 2. Sociodemographic characteristics of the Nielsen Homescan Consumer panel in 2018 projected to the Australian population compared with 2016 Australian Census data.**

| **Characteristic** | **Nielsen Homescan Consumer Panel 2018** | | **Australian Census 2016** | |
| --- | --- | --- | --- | --- |
|  | **Classification** | **N^1^ (%)** | **Classification** | **N (%)** |
| **Household size** | 1  2  3  4  5+ | 1,579 (22.0)  2,499 (34.8)  1,230 (17.1)  1,188 (16.5)  692 (9.6) | 1  2  3  4  5+ | 2,023,542 (24.4)  2,768,290 (33.4)  1,338,366 (16.2)  1,313,553 (15.9)  842,333 (10.2) |
| **Number of households in each state** | New South Wales  Victoria  Queensland  South Australia  Western Australia  Tasmania  Northern Territory | 2,367 (32.9)  1,824 (25.4)  1,435 (20.0)  621 (8.6)  711 (9.9)  182 (2.5)  48 (0.7) | New South Wales  Victoria  Queensland  South Australia  Western Australia  Tasmania  Northern Territory | 3,178,191 (33.2)  2,405,680 (25.1)  1,947,306 (20.4)  714,845 (7.5)  1,008,271 (10.5)  221,327 (2.3)  90,362 (0.9) |
| **Annual household gross income** | Low ($0 - $45,000)  Middle ($45,001 - $100,000)  High ($100,000+) | 2,277 (31.7)  2,843 (39.6)  2,068  (28.8) | Low ($0 - $41,599)  Middle ($41,600 - $103,999)  High ($104,000+) | 2,179,100 (24.0)  3,191,200 (35.7)  3,589,000 (40.2) |

^1^The number of households displayed are unweighted i.e. prior to survey weights applied to the dataset.

49

51

79

21

82

18

49

51

44

56

8

92

41

59

63

37

49

51

41

59

0

20

40

60

80

100

Percentage of products meeting / not meeting targets (%)

10

9

8

7

6

5

4

3 (Retailer)

2 (Retailer)

1 (Retailer)

Meeting target

Not meeting target

**Additional Figure 2. Percentage of products meeting and not meeting proposed Healthy Food Partnership sodium targets**. The food companies displayed are those that were ranked as the top 10 contributors to total sodium purchases made by Australian households. The number of products meeting/not meeting targets were calculated using both the FoodSwitch and Nielsen Homescan Panel and reflect the total volume of products sold by each company for which a proposed HFP target has been assigned.

**Additional Table 3. Sodium from Australian household packaged food and beverage purchases according to income level**

| **Category** | **Income level** | **Weight of food and beverages (g/d per capita)**  **Mean^1^** | **Sodium (mg/d per capita)** | | **Mean purchase-weighted sodium content (mg/100g)^2^** |
| --- | --- | --- | --- | --- | --- |
|  |  |  | **Mean^1^** | **Median**  **(25th to 75th percentiles)** |  |
| Foods | Low | 419 | 1224^a^ | 1104 (749 - 1571) | 388 |
|  | Middle | 360 | 1075^b^ | 968 (658 - 1354) | 401 |
|  | High | 302 | 920^c^ | 851 (556 - 1158) | 411 |
|  |  |  |  |  |  |
| Beverages | Low | 335 | 89^a^ | 69 (33 - 122) | 24 |
|  | Middle | 283 | 73^b^ | 56 (29 - 95) | 24 |
|  | High | 236 | 61^c^ | 45 (25 - 81) | 24 |
|  |  |  |  |  |  |
| Table salt | Low | 1.06 | 387^a^ | 68 (0 - 506) | 36140 |
|  | Middle | 0.81 | 296^b^ | 63 (0 - 380) | 35817 |
|  | High | 0.73 | 267^c^ | 63 (0 - 338) | 35977 |
|  |  |  |  |  |  |
| Total | Low | 755 | 1700^a^ | 1436 (958 - 2125) | 481 |
|  | Middle | 644 | 1444^b^ | 1279 (848 - 1777) | 479 |
|  | High | 539 | 1248^c^ | 1085 (740 - 1562) | 481 |

Note: Low = $954 per week or less per household, Middle = $955 - $2,000 per week per household, High = $2469 per week per household. ^1^ Standard error (SE) for weight of products purchased (g/d per capita) and sodium (mg/d per capita) not displayed as SE ≤0.1 for each mean value. ^a,b,c^ Within each category, mean values with unlike superscript letters indicate mean sodium (mg/d per capita) is significantly different (*P*<0.01). Significance tested using one-way ANOVA. Post-hoc significance tested using Tukey's honest significance test. ^2^Purchase-weighted sodium content (mg/100g): weight of sodium (mg) divided by the total weight (g) of products purchased (package size x quantity sold in 2018).

**Additional Table 4. Sodium acquired from Australian household food and beverage purchases, including barcoded and non-barcoded products**

|  | **Weight of products purchased**  **(g/d per capita)**  **Mean^1^** | **Sodium (mg/d per capita)** | | **Contribution to total weight of sodium purchases (%)** |
| --- | --- | --- | --- | --- |
| **Category** |  | **Mean^1^** | **Median (25th to 75th percentiles)** |  |
| Foods | 583 | 1292 | 1166 (814 - 1599) | 77 |
| Beverages | 284 | 74 | 57 (30 – 99) | 4 |
| Table salt | 1 | 317 | 84 (0 – 380) | 18 |
| Total | 868 | 1683 | 1458 (1016– 2082) | 100 |

^1^Standard error (SE) for weight of products purchased (g/d per capita) and sodium (mg/d per capita) not displayed as SE ≤0.4 for each mean value.
